# Identifying a Novel Role for Fractalkine (CX3CL1) in Memory CD8^+^ T Cell Accumulation in the Omentum of Obesity-Associated Cancer Patients

**DOI:** 10.3389/fimmu.2018.01867

**Published:** 2018-08-13

**Authors:** Melissa J. Conroy, Stephen G. Maher, Ashanty M. Melo, Suzanne L. Doyle, Emma Foley, John V. Reynolds, Aideen Long, Joanne Lysaght

**Affiliations:** ^1^Department of Surgery, St. James’s Hospital, Trinity College Dublin, Trinity Translational Medicine Institute, Dublin, Ireland; ^2^School of Biological Sciences, Dublin Institute of Technology, Dublin, Ireland; ^3^Gastro-Intestinal Medicine and Surgery, St. James’s Hospital, Dublin, Ireland; ^4^Department of Clinical Medicine, St. James’s Hospital, Trinity College Dublin, Trinity Translational Medicine Institute, Dublin, Ireland

**Keywords:** fractalkine, CX3CR1, T cells, obesity, upper gastrointestinal cancer, inflammation, adhesion, omentum

## Abstract

The omentum is enriched with pro-inflammatory effector memory CD8^+^ T cells in patients with the obesity-associated malignancy, esophagogastric adenocarcinoma (EAC) and we have identified the chemokine macrophage inflammatory protein-1alpha as a key player in their active migration to this inflamed tissue. More recently, others have established that subsets of memory CD8^+^ T cells can be classified based on their surface expression of CX3CR1; the specific receptor for the inflammatory chemokine fractalkine. CD8^+^ T cells expressing intermediate levels (CX3CR1^INT^) are defined as peripheral memory, those expressing the highest levels (CX3CR1^HI^) are effector memory/terminally differentiated and those lacking CX3CR1 (CX3CR1^NEG^) are classified as central memory. To date, the fractalkine:CX3CR1 axis has not been examined in the context of CD8^+^ T cell enrichment in the omentum and here we examine this chemokines involvement in the accumulation of memory CD8^+^ T cells in the omentum of EAC patients. Our data show that fractalkine is significantly enriched in the omentum of EAC patients and drives migration of T cells derived from EAC patient blood. Furthermore, CX3CR1 is endocytosed specifically by CD8^+^ T cells upon encountering fractalkine, which is consistent with the significantly diminished frequencies of CX3CR1^INT^ and CX3CR1^HI^ CD8^+^ T cells in the fractalkine-rich environment of omentum in EAC, relative to matched blood. Fractalkine-mediated endocytosis of CX3CR1 by CD8^+^ T cells is sustained and is followed by enhanced surface expression of L-selectin (CD62L). These novel data align with our findings that circulating CX3CR1^NEG^ CD8^+^ T cells express higher levels of L-selectin than CX3CR1^INT^ CD8^+^ T cells. This is consistent with previous reports and implicates fractalkine in the conversion of CX3CR1^INT^ CD8^+^ T cells to a CX3CR1^NEG^ phenotype characterized by alterations in the migratory capacity of these T cells. For the first time, these findings identify fractalkine as a driver of T cell migration to the omentum in EAC and indicate that CD8^+^ T cells undergo sequenced fractalkine-mediated alterations in CX3CR1 and L-selectin expression. These data implicate fractalkine as more than a chemotactic cytokine in obesity-associated meta-inflammation and reveal a role for this chemokine in the maintenance of the CX3CR1^NEG^ CD8^+^ T cell populations.

## Introduction

The flexibility and multifaceted functionality of chemokines together with their involvement in multiple inflammatory diseases has ignited an interest in their potential as targets for immunotherapy (1, 2). Indeed, there has been a paradigm shift in the classification of chemokines as solely chemotactic cytokines and emerging evidence has uncovered their additional functions in the generation of effector and memory T cells and the co-stimulation of cytokine secretion by T cells (3–5). Most recently, the specific receptor for the inflammatory chemokine fractalkine, CX3CR1 has been used to classify memory CD8^+^ T cell subsets (5).

Fractalkine (CX3CL1) exists in both a soluble and transmembrane form, and plays a role in both immune cell migration and adhesion and has been implicated in multiple inflammatory diseases such as asthma, dermatitis, diabetes, and neuropathic pain (6–12). The involvement of this chemokine in several inflammatory disorders is facilitated through its induction by inflammatory cytokines such as TNF-α, IFN-γ, and IL-1 (13). In the context of obesity, fractalkine has already been implicated in obesity-associated diseases such as diabetes and cardiovascular disease and in the chronic inflammation that precedes metabolic dysfunction; meta-inflammation (7, 8, 10, 14). Fractalkine is known to be expressed by endothelial cells and facilitates vascular recruitment and adhesion of macrophages, NK cells, and T cells, however, its expression by both the stromal vascular fraction (SVF) and the adipocyte fraction has been demonstrated in the adipose tissue (8). Most studies have focused on the role of fractalkine in macrophage-mediated adipose tissue inflammation (8, 15, 16), with previous work demonstrating a role for fractalkine in macrophage recruitment and macrophage-adipocyte adhesion in adipose tissue but, to our knowledge there are no reports of fractalkine-driven migration of T cells in obesity (8).

The omentum forms the largest component of the visceral adipose tissue (VAT) compartment and is enriched with leukocyte aggregates which liken it to the follicles of secondary lymphoid tissues and promote immune responses and inflammation (17). We have previously shown that the omentum is a hot bed of T cell-mediated inflammation in patients with esophagogastric adenocarcinoma (EAC), a malignancy that arises in a background of inflammation and importantly has one of the strongest associations with obesity of all malignancies (18). Furthermore, we have identified the macrophage inflammatory protein-1alpha (MIP-1α)/CCR1 axis as a key pathway governing T cell migration to the omentum of EAC patients and reported its therapeutic potential in the space of obesity-associated inflammation (19). However, the fractalkine pathway may present a more attractive and more specific therapeutic target in this setting as there is only one known receptor for this chemokine (CX3CR1), in contrast to the redundancy observed in other chemokine pathways (1, 20, 21). We have already demonstrated that the omentum of EAC patients is primed for fractalkine-mediated inflammation through an abundance of TNF-α-producing CD8^+^ and CD4^+^ T cells and secreted IL-1β and here, we address the role of fractalkine in inflammatory and cytotoxic T cell trafficking and retention in this inflamed tissue (22, 23). These questions are extremely relevant at a time when the global obesity problem has reached epidemic proportions and shows no signs of abating (www.who.int).

For the first time, this study identifies a role for fractalkine in the recruitment of T cells to the inflamed omentum of EAC patients implicating this chemokine as a player and potential therapeutic target in pathological T cell-mediated inflammation in obesity and obesity-associated malignancies. Importantly, our data also reveal fractalkine-mediated regulation of CX3CR1 and L-selectin expression by CD8^+^ T cells, placing this inflammatory chemokine as an orchestrator of cytotoxic T cell trafficking and memory in EAC. The data generated here has ramifications for fractalkine as a therapeutic target for inflammation particularly in the context of cancer indicating that antagonism of this pathway might have consequences for L-selectin-dependent cytotoxic T cell homing to lymph nodes and anti-tumor immunity.

## Materials and Methods

### Subjects

Forty-seven consecutive consenting patients with EAC, attending the National Esophageal and Gastric Center at St. James’s Hospital, Dublin from 2011 to 2018 were enrolled in this study. The patient cohort was similar in age and ethnicity, and 81% had received neoadjuvant chemo-radiotherapy. The patient group included 37 males and 10 females, representative of the male predominance in EAC, with an average age of 65.9 years. The mean BMI at time of surgery was 26.4 and CT-defined visceral fat area (VFA) was 126.54 cm^2^ (Table 1). Control blood and omentum were taken from a group of non-cancer control patients attending St. James’s Hospital, Dublin for laparoscopic cholecystectomy. All cancer patients were evaluated by a dietician. Body mass index, waist circumference, and anthropometric variables were measured as described previously (23). VFA was assessed by computer tomography as previously described, with more than 160 and 80 cm^2^ defining visceral obesity in males and females, respectively (24). Serum C-reactive protein (CRP) levels were determined as part of routine testing at time of surgery. Metabolic syndrome was measured as per the IDF definition (25).

**Table 1:** Demographic data.

Age (range years)	65.9 (35–93)
Sex ratio (M:F)	37:10
Esophageal adenocarcinoma	38
Gastric adenocarcinoma	9
Tumor stage^a^	
T0	4
T1	11
T2	8
T3	20
T4	1
Nodal status^a^	
Positive	21
Negative	23
Mean BMI (kg/m^2^) (range)^b^	26.4 (17.8–36.4)
Underweight (BMI < 19.9)	4
Normal weight (BMI 20–24.9)	12
Overweight (BMI 25–29.9)	13
Obese (BMI > 30)	14
Mean waist circumference (cm) (range)	93.8 (68–115)
Centrally obese by waist circumference^c^	57.75%
Mean visceral fat area (VFA) (cm^2^) (range)	126.54 (4.65–353.05)
Viscerally obese by VFA^d^	30%
Received Neoadjuvant CRT	81%

*^a^Tumor stage and nodal status was not available for three patients*.

*^b^BMI was not available for four patients*.

*^c^Obese waist circumference ≥94 cm for men and ≥80 cm for women (25)*.

*^d^Obese VFA >160 cm^2^ for men and >80 cm^2^ for women (24)*.

### Ethics Approval Statement

The work was performed in accordance with The Code of Ethics of the World Medical Association (Declaration of Helsinki) for experiments involving humans. Patients provided informed consent for sample and data acquisition and the study received full ethical approval from the St. James’s Hospital Ethics Review Board. Patient samples were pseudonymized to protect the privacy rights of the patients.

### Sample Preparation

Peripheral blood mononuclear cells (PBMC) were isolated by density centrifugation using Ficoll-Paque™ Plus (GE Healthcare, Uppsala, Sweden). Omental samples (10 g from each patient) were digested enzymatically to obtain SVF as previously described (22, 23). Adipose tissue conditioned media (ACM) was prepared as previously described (22).

### Quantification of Soluble Fractalkine and CX3CR1 Levels in Serum and Omentum

The V-PLEX™ Fractalkine plate (Meso Scale Discovery) was used to detect the levels of fractalkine in the serum and ACM of 19 EAC patients according to the manufacturer’s instructions and read using an MSD Sector S600. The Human CX3CR1 ELISA Kit (ELISA Genie) was used to measure secreted CX3CR1 in the serum and ACM of 10 EAC patients and read using a VersaMax™ ELISA microplate reader.

### Examining Secreted Adhesion Molecule Levels in the Omentum of EAC Patients

The Evidence Investigator Adhesion Molecules Array (RANDOX) was used to detect the concentrations of VCAM-1, ICAM-1, L-selectin, P-selectin, and E-selectin in the ACM of five EAC patients according to the manufacturer’s instructions and read using a RANDOX Evidence Investigator.

### T Cell Chemotaxis Assays

To activate the T cells for chemotaxis, PBMC were prepared from the blood of five EAC patients and three non-cancer controls, and incubated with CD3/CD28 T-cell expander Dynabeads (Invitrogen) at a bead:cell ratio of 1:1. Following activation, the beads were removed and the T cells were counted and resuspended at densities of 2 × 10^6^ cells/ml serum-free RPMI media. Chemotaxis was assessed using a 5-µm pore transwell filter system (Corning Inc., USA). Cells were added to the top chamber in a volume of 100 µl RPMI, while the bottom chamber contained 600 µl of the following; M199 media alone (negative control) or M199 media supplemented with fractalkine at a concentration of 30 ng/ml (based on the mean concentration in EAC ACM) or ACM derived from four obese and three non-obese EAC patients or ACM derived from two obese and three non-obese non-cancer controls. The assay was incubated for 2 h at 37°C before removal of the insert. The media in the bottom chambers were taken and centrifuged at 1,300 RPM for 3 min and then stained for CD3 expression. CountBright Absolute Counting Beads (Invitrogen) were used to enumerate the absolute number of CD3^+^ T cells that had migrated.

### Quantification of CX3CR1 Expressing T Cells in Blood and Omentum and Phenotyping of CX3CR1^NEG^ and CX3CR1^INT^ CD8^+^ T Cell Populations

Freshly isolated PBMC and SVF were stained with monoclonal antibodies specific for human surface markers; PE-efluor-labeled CD3 and APC-eFluor780-labeled CD27 (eBioscience, Hatfield, UK), PE-Cy5.5-labeled CD4, APC-labeled ICAM-1 (CD54), V500-labeled CD45RA, and BV421-labeled CD8 (BD Biosciences, Oxford, UK), PE-Cy5-labeled L-selectin (Abcam, Cambridge, UK), and PE-labeled CX3CR1 (Miltenyi Biotec, Germany). Cells were acquired using CyAn ADP flow cytometer (Beckman Coulter) and analyzed with FlowJo software (TreeStar Inc.).

### CX3CR1 Surface Expression by T Cells Following Recombinant Fractalkine Treatment

To examine the effects of fractalkine on CX3CR1 expression by CD8^+^ T cells, PBMC from 17 EAC patients were seeded in RPMI media at 1 × 10^6^ cells/ml and treated with M199 media or 30 ng/ml of recombinant fractalkine for 15 min, 30 min, 1 h, 2 h, and 24 h and subsequently analyzed for CX3CR1 surface expression using flow cytometry, as described above. To test whether CX3CR1 was endocytosed, PBMC from 10 EAC patients were treated with M199 media (untreated) or 30 ng/ml of recombinant fractalkine for 2 h at 4°C (cold treatment) or in the presence of 80 µM of the GTPase inhibitor dynasore (Sigma Aldrich). To examine whether CX3CR1 is recycled to the surface of CD8^+^ T cells following fractalkine treatment, PBMC from three donors were seeded in RPMI media at 1 × 10^6^ cells/ml and treated with M199 media alone or 30 ng/ml of recombinant fractalkine for 2 h. Following 2 h, cells were washed and placed in fractalkine-free media for 24 and 48 h or placed in fractalkine-free media supplemented with 50 ng/ml of TNF-α or 100 ng/ml of IFN-γ, IL-6, IL-7, IL-17, IL-18, IL-15, or IL-36 or 50 U/ml of IL-2 for 24 h and subsequently analyzed for CX3CR1 surface expression using flow cytometry.

### Examining CX3CR1 Protein Expression Following Treatment With Recombinant Fractalkine

CD8^+^ T cells from three healthy control subjects were isolated from PBMC using the EasySep™ Human CD8^+^ T Cell Isolation Kit (Stemcell Technologies) and subsequently seeded at a density of 1 × 10^6^ cells/ml RPMI media and treated with 30 ng/ml of fractalkine for 24 and 48 h. Flow cytometry was used to confirm reduction of CX3CR1 surface expression on a subset of treated and untreated cells after 24 h and the remainder of the cells were lysed. Protein was extracted using a 6 M urea lysis buffer (15 min on ice). Protein was quantified against a standard curve using the Pierce BCA Protein Assay Kit (Thermofisher). Protein (60 µg per sample) was loaded onto a 10% SDS page gel and electrophoresed for 80 min. Separated protein was subsequently transferred to a PVDF membrane. Membrane was blocked in 0.5% Bovine Serum Albumin (in TBST) for 1.5 h at room temperature. The membrane was incubated for 1.5 h at room temperature with mouse anti-human CX3CR1 antibody (H-70) (Santa Cruz) at 1:1,000 dilution in 0.5% BSA TBST. The membrane was incubated for 1 h at room temperature with secondary antibody (goat anti-mouse IgG-HRP at 1:5,000 dilution in 0.5% BSA TBST). Bands were visualized using the West Pico chemiluminescent substrate on a gel doc. Beta-actin primary mouse anti-human at 1:10,000 dilution (Santa Cruz) was used as a loading control. Protein extract quality was confirmed visually *via* coomassie blue staining (10% gel, 20 µg protein per sample). CD8^+^ T cells from three control subjects were isolated from PBMC using the EasySep™ Human CD8^+^ T Cell Isolation Kit (Stemcell Technologies) and subsequently seeded in RPMI media at 1 × 10^6^ cells/ml and treated with 30 ng/ml of fractalkine for 24 and 48 h. Cell supernatant was collected after 24 and 48 h and the Human CX3CR1 ELISA Kit (ELISA Genie) was used to compare secreted CX3CR1 in the untreated and fractalkine-treated cells.

### Assessing Integrin and Adhesion Molecule Expression Together With Memory Phenotype of CD8^+^ T Cells Following Fractalkine Treatment

To examine the effects of fractalkine on CX3CR1 expression by CD8^+^ T cells, PBMC from six EAC patients were treated with M199 media alone or M199 media supplemented with 30 ng/ml of recombinant fractalkine for 24 h and subsequently analyzed for VLA-4, LFA-1, alpha4 integrin, beta7 integrin, ICAM-1, L-selectin, CD45RA, and CD27 surface expression using flow cytometry, as described above.

### Statistical Analyses

Statistical analysis was carried out using Prism GraphPad Version 5.0. Differences between groups were assessed using two-tailed paired, Wilcoxon sign-rank test, unpaired non-parametric Mann–Whitney *U* tests, and one-way ANOVA with Tukey *post hoc* analysis where appropriate. Significant associations between fractalkine, CX3CR1, and clinical parameters were investigated using Spearman’s rank-order correlation test. *p* Values of <0.05 were considered to be significant.

## Results

### Significantly High Levels of Soluble Fractalkine in the Omentum of EAC Patients Can Drive Migration of EAC Patient-Derived T Cells

**Figure 1:**
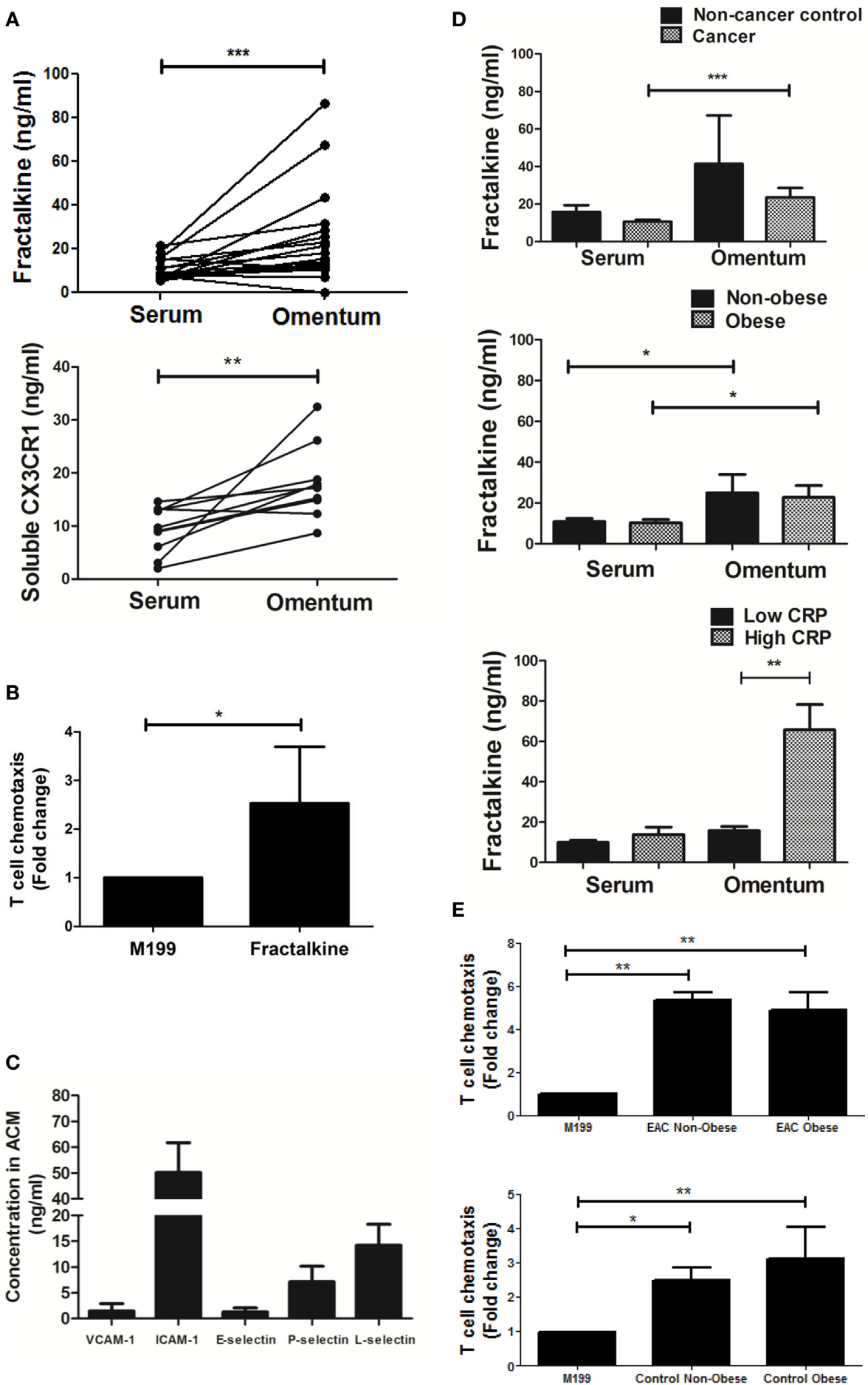
Both soluble fractalkine and CX3CR1 are secreted in abundance in the omentum of esophagogastric adenocarcinoma (EAC) patients and fractalkine can drive migration of EAC patient-derived T cells. **(A)** Scatter plots showing the fractalkine (top) and soluble CX3CR1 levels (bottom) in paired samples of serum and adipose tissue conditioned media (ACM) from 10 to 19 EAC patients. **(B)** Bar charts showing significant fold change migration of peripheral blood-derived T cells from five EAC patients to M199 media supplemented with 30 ng/ml of recombinant fractalkine, relative to M199 alone negative control. **(C)** Bar charts showing the levels of soluble VCAM-1, ICAM-1, E-selectin, P-selectin, and L-selectin in omental adipose tissue conditioned media (ACM) from five EAC patients. **(D)** Bar charts showing the fractalkine levels in serum and ACM from 3 non-cancer control subjects and 20 EAC patients, from 9 non-obese and 11 obese EAC patients, and 17 EAC patients with serum C-reactive protein (CRP) <5 mg/l (low CRP) and 3 EAC patients with serum CRP >5 mg/l (high CRP). **(E)** Bar charts showing significant fold change migration of peripheral blood-derived T cells to ACM from three non-obese and four obese EAC patients and three non-obese and two obese non-cancer controls. *p < 0.05, **p < 0.01, ***p < 0.001 by paired, unpaired *t* tests and one-way ANOVA with Tukey *post hoc* analysis.

Secreted fractalkine was quantified by MSD V-Plex ELISA in the matched serum and omental adipose tissue conditioned media (ACM) of 19 EAC patients revealing that levels of this chemokine were significantly higher in ACM (mean: 23.66 ng/ml) compared to serum (mean: 10.56 ng/ml) (*p* < 0.001, Figure 1A). Soluble CX3CR1 ELISA revealed that the soluble form of the fractalkine receptor was also significantly abundant in the ACM of 10 EAC patients, compared to matched serum suggesting that the fractalkine receptor can be shed in the omental microenvironment (mean: 18.1 versus 9.3 ng/ml, *p* < 0.01, Figure 1A). To confirm whether T cells respond and migrate toward fractalkine-elicited chemotactic signals in EAC, patient-derived T cell chemotaxis to 30 ng/ml of fractalkine was measured using a transwell system. Significantly higher fold-levels of T cell migration toward recombinant fractalkine, compared to serum-free M199 media were observed supporting our hypothesis of fractalkine-driven T cell migration to omentum in EAC (M199 versus fractalkine fold change: 1 versus 2.456, *p* < 0.05, Figure 1B). An adhesion molecule array was used to screen omentum-derived ACM from five EAC patients for soluble adhesion molecules and ICAM-1 and L-selectin were shown to be most abundant, indicative of the adhesive properties of the omental microenvironment (Figure 1C). Secreted fractalkine levels were significantly higher in omentum compared to matched serum regardless of patient obesity status and interestingly, there were significantly higher levels of soluble fractalkine in the omentum of EAC patients with elevated serum CRP, i.e., >5 mg/l compared to those with lower serum CRP, i.e., <5 mg/l (mean: 65.8 versus 13.87 ng/ml, *p* < 0.05) (Figure 1D; Table 2). Furthermore, our data show significant levels of T cell migration to ACM in both non-cancer and EAC patients, irrespective of obesity status (Figure 1E).

**Table 2:** Correlations of CX3CL1 levels and frequencies of CX3CR1^NEG^ expressing T cells with waist circumference, visceral fat area (VFA), and body mass index.

*R*^2^ values for correlations of CX3CR1 and the following measurements	Waist circumference *R*^2^ value	VFA *R*^2^ value	Body mass index *R*^2^ value
Serum CX3CL1	0.028	0.058	0.119
Omental CX3CL1	0.003	0.126	0.134
Peripheral CX3CR1^+^ CD4^+^ T cells	−0.15	0.008	−0.054
Omental CX3CR1^+^ CD4^+^ T cells	0.193	0.159	0.036074
Peripheral CX3CR1^+^ CD8^+^ T cells	−0.298	−0.216	−0.164
Omental CX3CR1^+^ CD8^+^ T cells	0.464	0.044	0.073

### Low Frequencies of CX3CR1^+^ CD8^+^ T Cells in the Omentum of EAC Patients Are due to Significantly Diminished Frequencies of Both the CX3CR1^INT^ and CX3CR1^HI^ Subsets of CD8^+^ T Cells

**Figure 2:**
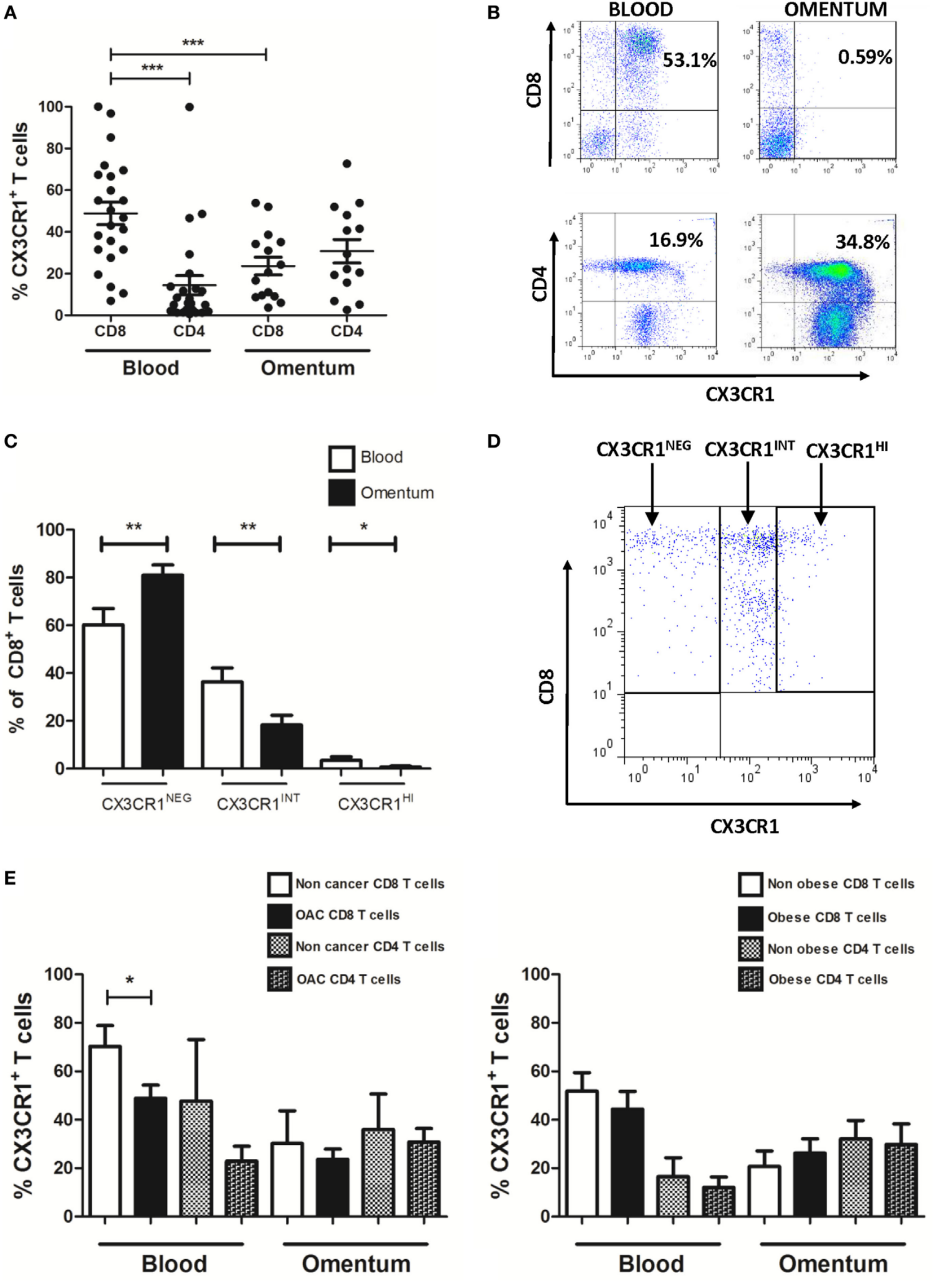
Frequencies of CX3CR1^+^ CD8^+^ T cells are significantly lower in omentum of esophagogastric adenocarcinoma (EAC) patients compared to frequencies of both peripheral blood CX3CR1^+^ CD8^+^ T cells and CX3CR1^+^ CD4^+^ T cells. Peripheral blood mononuclear cells and stromal vascular fraction of omentum were isolated from EAC patients. **(A)** Scatterplots show the frequencies of CX3CR1^+^ CD8^+^ and CD4^+^ T cells in the blood and omentum of a total of 24 EAC patients. **(B)** Representative dot plots of CX3CR1^+^ CD8^+^ and CX3CR1^+^ CD4^+^ T cells (gated on and shown as a percentage of total CD3^+^ population) in blood and omentum. **(C)** Bar chart showing the frequencies of CD8^+^ cells expressing no CX3CR1 (CX3CR1^NEG^), intermediate (CX3CR1^INT^), and high levels of CX3CR1 (CX3CR1^HI^) in the blood (white) and omentum (black) of 24 EAC patients. **(D)** Representative dot plot of circulating CD8^+^ T cells expressing no CX3CR1 (CX3CR1^NEG^), intermediate levels of CX3CR1 (CX3CR1^INT^), and high levels of CX3CR1 (CX3CR1^HI^). **(E)** Bar charts showing the CX3CR1^+^ CD8^+^ and CD4^+^ T cells in the blood and omentum of 8 non-cancer control subjects and 24 EAC patients (left) and 14 non-obese and 10 obese EAC patients (right). *p < 0.05, *p < 0.01, ***p < 0.001 by paired and unpaired *t* tests.

The frequencies of CD4^+^ and CD8^+^ T cells expressing the chemokine receptor CX3CR1 were examined in the blood and omentum of a total of 24 EAC patients by flow cytometry. Our results identified significantly higher frequencies of CX3CR1^+^ CD8^+^ T cells in the circulation compared to their CD8^+^ counterparts in omentum (blood versus omentum mean: 48.87 versus 23.64%, *p* = 0.0002, Figure 2A) and compared to proportions of circulating CX3CR1^+^ CD4^+^ T cells (mean: 14.48%, *p* < 0.0001, Figure 2A). These differences were paralleled by substantially but not significantly higher frequencies of CX3CR1^+^ CD4^+^ T cells in EAC omentum (mean: 30.76%, Figures 2A,B) compared to blood. Further analysis revealed that the diminished numbers of CX3CR1^+^ CD8^+^ T cells within the omentum were due to significantly lower frequencies of CD8^+^ T cells expressing both intermediate and high levels of CX3CR1 and significantly higher frequencies of CX3CR1^NEG^ populations in omentum, compared to blood (blood versus omentum mean of CX3CR1^NEG^: 60.24 versus 81.03%, *p* < 0.01, CX3CR1^INT^: 36.34 versus 18.25%, *p* < 0.01, CX3CR1^HI^: 3.41 versus 0.72%, *p* < 0.05, Figure 2C). The highest proportions of circulating CX3CR1^+^ cells were identified within the CX3CR1^INT^ CD8^+^ T cell population (Figures 2C,D). While such observations in CX3CR1 expression by T cells were consistent among obese and non-obese EAC patients, there were significantly higher frequencies of CX3CR1^+^ CD8^+^ T cells in the circulation of non-cancer controls, compared to EAC patients (non-cancer versus EAC: 70.33 versus 48.87%, *p* < 0.05, Figure 2E; Table 2).

### CX3CR1 Expression by Peripheral Blood but Not Omental CD8^+^ T Cells Is Significantly Diminished Following Treatment With Recombinant Fractalkine

**Figure 3:**
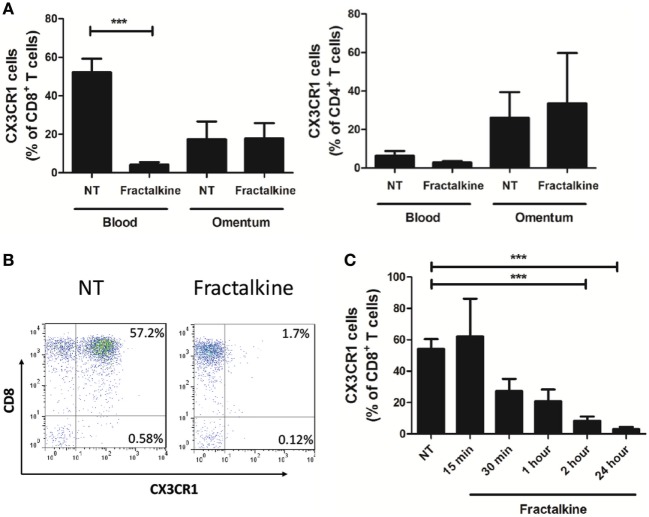
Peripheral blood CD8^+^ T cells exhibit a significant and sustained reduction in CX3CR1 surface expression following treatment with recombinant fractalkine. **(A)** Frequencies of CX3CR1^+^ cells, as a percentage of CD8^+^ T cells (left) and CD4^+^ T cells (right) following; treatment with M199 media alone (NT) or 30 ng/ml of recombinant fractalkine for 2 h (*n* = 17); **(B)** representative dot plots of CX3CR1 expression following treatment with M199 media alone (NT) or 30 ng/ml of recombinant fractalkine for 2 h. **(C)** Frequencies of CX3CR1^+^ cells, as a percentage of CD8^+^ T cells following treatment with M199 media alone (NT) or 30 ng/ml of recombinant fractalkine across a time course of 15 min, 30 min, 1 h, 2 h, or 24 h (*n* = 4). ***p < 0.001 by paired *t* tests.

**Figure 4:**
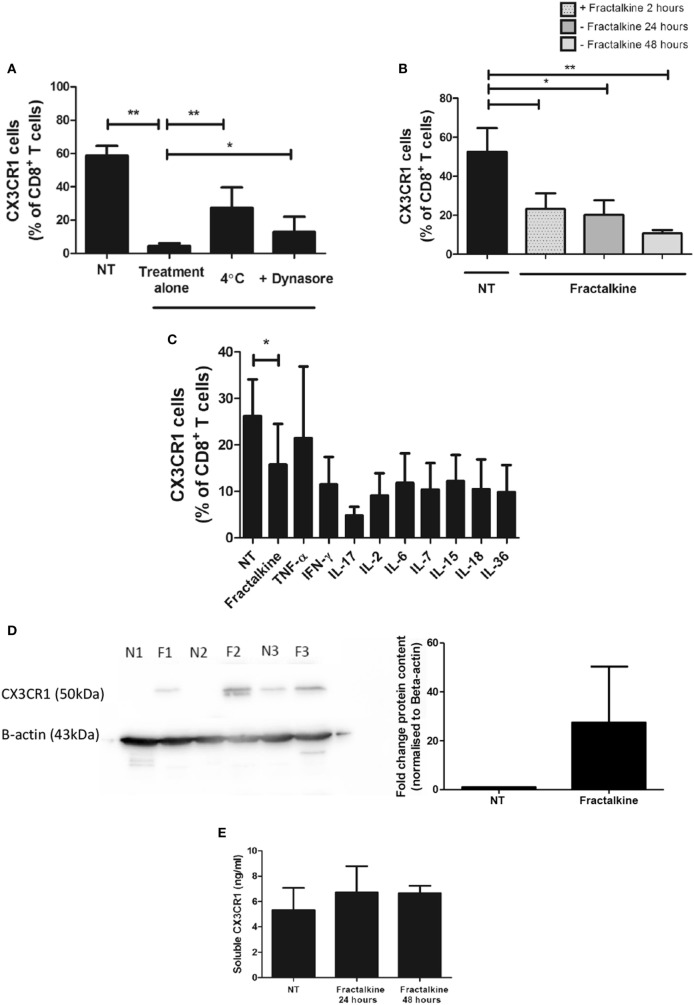
CX3CR1 is endocytosed following fractalkine treatment and is not subsequently recycled to the surface of CD8^+^ T cells or secreted but, intracellular accumulations of the protein are detectable. **(A)** Frequencies of CX3CR1^+^ cells, as a percentage of CD8^+^ T cells following treatment with M199 media alone (NT), 30 ng/ml recombinant fractalkine alone (treatment alone), 30 ng/ml recombinant fractalkine at 4°C and 30 ng/ml recombinant fractalkine plus 80 µM Dynasore (*n* = 10). **(B)** Bar chart showing frequencies of CX3CR1^+^ cells, as a percentage of CD8^+^ T cells following treatment with M199 media alone (NT) or 30 ng/ml of recombinant fractalkine for 24 h (fractalkine, light gray dot pattern) followed by removal from a fractalkine-free environment for 24 h (fractalkine, dark gray no pattern) or 48 h (fractalkine, light gray no pattern) (*n* = 6). **(C)** Bar chart showing frequencies of CX3CR1^+^ cells, as a percentage of CD8^+^ T cells following treatment with M199 media alone (NT) or 30 ng/ml of recombinant fractalkine for 24 h (fractalkine, light gray dot pattern) followed by removal from a fractalkine-free environment for 24 h and treatment with 50 ng/ml of TNF-α, 100 ng/ml of IFN-γ, IL-17, IL-6, IL-7, IL-15, IL-18, IL-36, and 50 U/ml of IL-2 (*n* = 3). **(D)** Western Blot (left) showing CX3CR1 and beta-actin protein in peripheral blood-derived CD8^+^ T cells from three donors following 24 h of no treatment (N1, N2, and N3) or fractalkine treatment (F1, F2, and F3) and densitometry data (right) from this western blot displayed as fold change bar chart. **(E)** Soluble CX3CR1 levels in the supernatant of peripheral blood mononuclear cells treated with M199 alone or fractalkine for 24 or 48 h (*n* = 3). *p < 0.05, **p < 0.01 by paired *t*-test.

To ascertain why enrichments of CX3CR1^+^ CD4^+^ T cells were detected in the omentum, while highest frequencies of CX3CR1^+^ CD8^+^ T cells were detected in the circulation, we assessed whether CX3CR1^+^ CD8^+^ T cells convert to CX3CR1^NEG^ CD8^+^ T cells upon encountering their ligand, which is secreted in abundance in the omental microenvironment. Blood-derived T cells from 17 EAC patients were treated with M199 media or recombinant fractalkine for 2 h to simulate the effects of the high fractalkine levels in the omental microenvironment. Flow cytometric analysis revealed that surface expression of CX3CR1 was significantly decreased on peripheral blood CD8^+^ but not CD4^+^ T cells or omental CD8^+^ T cells following 2 h treatment with recombinant fractalkine (untreated versus treated CD8^+^ T cells: 52.2 versus 4.238, *p* = 0.0001, Figures 3A,B). Further analysis revealed that CX3CR1 expression by peripheral blood CD8^+^ T cells is reduced between 15 and 30 min post-treatment with most significant reductions observed at 2 and 24 h post-treatment (Figure 3C, *p* < 0.001). Fractalkine treatments performed at a temperature of 4°C or in combination with treatment of 80 µM dynasore, a GTPase inhibitor, demonstrated significantly reduced efficacy to decrease CX3CR1 expression on CD8^+^ T cells thus confirming that the observed reductions were due to endocytosis (Figure 4A).

### CX3CR1 Internalization by CD8^+^ T Cells Is Sustained Following Treatment With Recombinant Fractalkine and the Receptor Is Not Degraded or Recycled to the Cell Surface

Surface expression of CX3CR1 by CD8^+^ T cells was not restored when 2 h treatment with fractalkine was followed by culture in fractalkine-free media for 24 and 48 h, suggesting that the receptor is not recycled to the surface following endocytosis (Figure 4B). Stimulation with 50 ng/ml of TNF-α or 100 ng/ml of IFN-γ, IL-6, IL-7, IL-17, IL-18, IL-15, or IL-36 or 50 U/ml of IL-2 for 24 h following removal of fractalkine, did not induce recycling of CX3CR1 to the surface of CD8^+^ T cells (Figure 4C). Western blot revealed that there are consistent accumulations of CX3CR1 protein levels in CD8^+^ T cells following 24 h fractalkine treatment compared to untreated CD8^+^ T cells demonstrating that the receptor is not degraded following fractalkine-mediated endocytosis (Figure 4D). Furthermore, ELISA revealed that the levels of CX3CR1 shed by T cells are not increased following 24 or 48 h of fractalkine treatment (Figure 4E).

### CX3CR1^NEG^ and CX3CR1^INT^ Populations of CD8^+^ T Cells in the Peripheral Blood Have Significantly Different Memory Phenotype and Adhesion Molecule Expression

**Figure 5:**
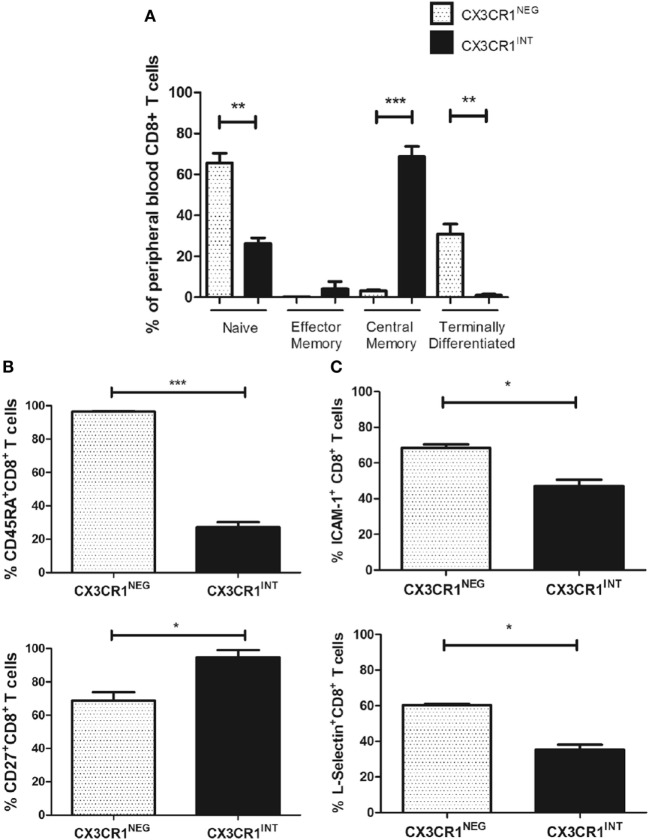
CX3CR1^NEG^ and CX3CR1^INT^ populations represent significantly different CD8^+^ T cells in the peripheral blood with significantly different memory phenotypes, L-selectin, and ICAM-1 expression. **(A)** Bar charts showing the frequencies of naive, central memory, effector memory, and terminally differentiated CD8^+^ T cells, characterized by CD45RA and CD27 expression within the CX3CR1^NEG^ and CX3CR1^INT^ populations in peripheral blood of six non-cancer controls. **(B)** Bar chart showing frequencies of peripheral blood-derived CD8^+^ T cells expressing CD45RA (top) and CD27 (bottom) within CX3CR1^NEG^ and CX3CR1^INT^ populations (*n* = 3). **(C)** Bar chart showing frequencies of peripheral blood-derived CD8^+^ T cells ICAM-1 and L-selectin within CX3CR1^NEG^ and CX3CR1^INT^ populations (*n* = 3). *p* < 0.05, **p < 0.01, ***p < 0.001 by unpaired *t* tests.

Since the CX3CR1^+^ compartments of CD8^+^ T cells were predominantly CX3CR1^INT^ and this subpopulation has been shown to have the potential to convert to CX3CR1^NEG^ CD8^+^ T cells, we focused our phenotypical comparisons between this subset and the CX3CR1^NEG^ CD8^+^ T cell subset [Figure 2C; (5)]. Significant differences in memory phenotype were identified between the CX3CR1^NEG^ and CX3CR1^INT^ CD8^+^ T cells based on their CD45RA and CD27 surface expression using the Dieli scheme; terminally differentiated (CD45RA^+^CD27^−^), naive (CD45RA^+^CD27^+^), effector memory (CD45RA^−^CD27^−^), and central memory (CD45RA^−^CD27^+^) (26). Our data revealed that circulating CX3CR1^INT^ CD8^+^ T cells were significantly different to CX3CR1^NEG^ CD8^+^ T cells in memory phenotype; CX3CR1^INT^ CD8^+^ T cells were predominantly central memory and effector memory phenotype while CX3CR1^NEG^ CD8^+^ T cells contained more naive and terminally differentiated cells (Figure 5A, central memory: *p* = 0.0001, effector memory: *p* < 0.05, naive: *p* = 0.001, terminally differentiated: *p* = 0.002). These phenotypical differences were attributable to significantly lower CD45RA surface expression and significantly higher CD27 surface expression within the CX3CR1^INT^ CD8^+^ T cell population (CX3CR1^INT^ versus CX3CR1^NEG^ CD45RA: 27.2 versus 96.4%, *p* < 0.001, CD27, 94.9 versus 68.8%, *p* < 0.05, Figure 5B). To elucidate whether our data compared to previous reports of lower L-selectin surface expression within CX3CR1^INT^ T cell population, we examined the expression of L-selectin and ICAM-1 on circulating CX3CR1^NEG^ and CX3CR1^INT^ CD8^+^ T cells (5). Our data revealed significantly lower frequencies of ICAM-1^+^ and L-selectin^+^ cells within the CX3CR1^INT^ CD8^+^ T cell population compared to their CX3CR1^NEG^ counterparts, indicating differential migratory and adhesive properties of these subsets (CX3CR1^NEG^ versus CX3CR1^INT^ ICAM-1: 68.4 versus 47.1%, *p* = 0.02, L-selectin: 60.4 versus 35.4%, *p* = 0.02, Figure 5C).

### Increased L-Selectin Surface Expression Follows Fractalkine-Mediated CX3CR1 Endocytosis on CD8^+^ T Cells

**Figure 6:**
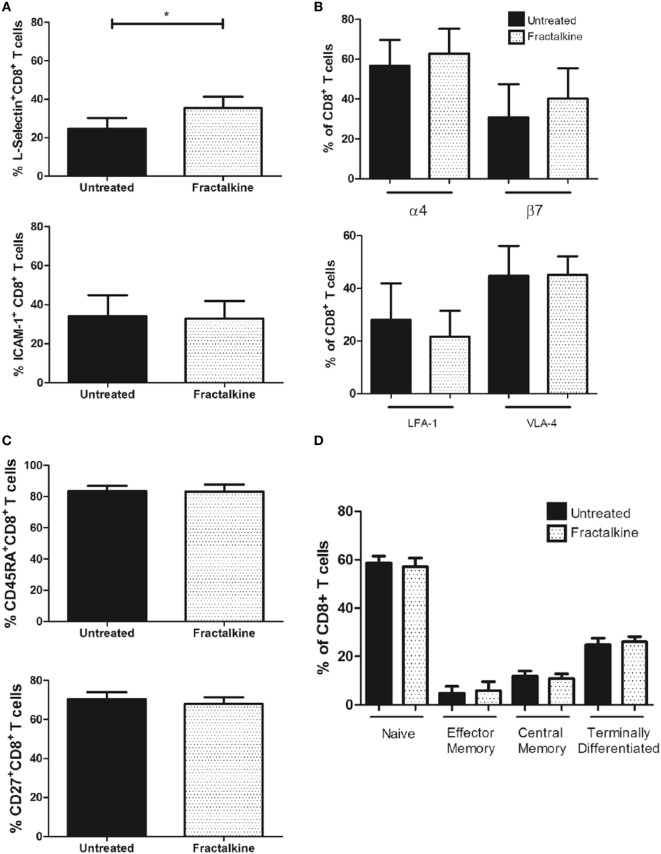
The fractalkine-mediated CX3CR1^INT^ to CX3CR1^NEG^ conversion of CD8^+^ T cells is followed by enhanced L-selectin expression. **(A)** Bar charts showing frequencies of peripheral blood-derived CD8^+^ T cells expressing L-selectin (top) and ICAM-1 (bottom) following treatment with M199 media alone (untreated, black) or 30 ng/ml of recombinant fractalkine for 24 h (fractalkine, white pattern) (*n* = 6). **(B)** Bar chart (top) showing frequencies of peripheral blood-derived CD8^+^ T cells expressing alpha4 and beta7 integrins following treatment with M199 media alone (untreated, black) or 30 ng/ml of recombinant fractalkine for 24 h (fractalkine, white pattern) (*n* = 6). **(B)** Bar chart (bottom) showing frequencies of peripheral blood-derived CD8^+^ T cells expressing LFA-1 and VLA-4 following treatment with M199 media alone (untreated, black) or 30 ng/ml of recombinant fractalkine for 24 h (fractalkine, white pattern) (*n* = 6). **(C)** Bar chart showing the frequencies of total CD45RA^+^ and CD27^+^ populations of CD8^+^ T cells following treatment with M199 media alone (untreated, black) or 30 ng/ml of recombinant fractalkine for 24 h (fractalkine, white pattern) (*n* = 6). **(D)** Bar chart showing the frequencies of naive, effector memory, central memory, and terminally differentiated CD8^+^ T cells, characterized by CD45RA and CD27 expression following treatment with M199 media alone (untreated, black) or 30 ng/ml of recombinant fractalkine for 24 h (fractalkine, white pattern) (*n* = 6). *p < 0.05 by paired *t* tests.

To simulate the high fractalkine levels in the omental microenvironment and examine whether this affects adhesion molecule expression and memory phenotype; fractalkine treatment of peripheral blood T cells from three donors was performed for 24 h. Fractalkine treatment induced a significant increase in surface expression of L-selectin but not ICAM-1 on CD8^+^ T cells following 24 h, indicating that it alters their adhesiveness and lymphoid tissue homing capacity (L-selectin: 24.6 versus 35.5%, *p* < 0.05, Figure 6A). Fractalkine treatment had no significant effects on ICAM-1, LFA-1, VLA-4, or integrin alpha4 and beta7 surface expression or memory phenotype of CD8^+^ T cells defined by CD45RA and CD27 expression (Figures 6B–D). In data not shown, 24 h fractalkine treatment had no significant effects on pro-inflammatory cytokine production by the CD8^+^ T cells.

## Discussion

### High Levels of Soluble Fractalkine in Omentum Can Drive T Cell Migration and May Represent a Hallmark of Meta-Inflammation in Obesity-Associated Cancer Patients

For the first time, this study has revealed significantly high levels of soluble fractalkine in the omental adipose tissue conditioned media (ACM) derived from patients with the obesity-associated cancer EAC. This is in line with previous observations of fractalkine enrichment in VAT (8). Known inducers of fractalkine include the inflammatory cytokines IFN-γ, TNF-α, and IL-1β (13). Such an abundance of fractalkine was unsurprising as we have reported enrichments of IFN-γ and TNF-α producing T cells together with secreted IL-1β in the omentum of EAC patients (13, 22, 23). Interestingly, significantly higher fractalkine levels were observed in the patients with highest serum CRP levels, which is indicative of its role in meta-inflammation and in line with previous reports of the circulating levels of CRP and fractalkine in metabolic dysfunction (7, 27). However, this is the first reported association of high fractalkine in the omentum and high serum CRP in obesity-associated cancer. Soluble fractalkine levels were significantly high in both obese and non-obese omentum in both cancer and non-cancer subjects. Furthermore, soluble cues from both obese and non-obese and both cancer and non-cancer omental microenvironments drove T cell migration suggesting that this tissue is a rich source of this chemokine and recruits significant numbers of T cells, regardless of obesity status. Interestingly, while fractalkine was secreted in abundance in the omentum and could drive *in vitro* EAC-derived T cell migration, this was not paralleled by high frequencies of T cells expressing its specific receptor in this tissue. In fact, the highest proportions of CX3CR1^INT^ cells were identified in circulating CD8^+^ T cell populations and not the omentum. Such frequencies were still significantly lower than CD8^+^ T cell frequencies in non-cancer controls suggesting that the fractalkine:CX3CR1 axis might be perturbed by malignancy in this study cohort. Together with our previously reported data, our investigations suggest that fractalkine together with MIP-1α in the omentum recruit inflammatory T cells to this tissue in EAC, most likely contributing to the pathological adipose tissue inflammation at the expense of effective anti-tumor immunity (19). As other studies have shown that fractalkine serves distinctive roles in different inflammatory diseases such as diabetes, dermatitis, asthma, and neuropathic pain, we propose that it may also serve functions additional to T cell chemotaxis in the omentum of EAC patients (8, 11, 12, 28).

### Diminished Frequencies of CX3CR1^+^ CD8^+^ T Cells and Increased Frequencies of CX3CR1^NEG^ CD8^+^ T Cells in Omentum Are Indicative of Fractalkine-Mediated CX3CR1^INT^ to CX3CR1^NEG^ Conversion

Our data reveal significantly higher proportions of CX3CR1^HI^ and CX3CR1^INT^ CD8^+^ T cells in the peripheral blood of EAC patients and significantly diminished frequencies in the omentum. CX3CR1^HI^ and CX3CR1^INT^ CD8^+^ T cells represent two of three distinct memory CD8^+^ T cell subsets which have recently been defined by their CX3CR1 expression (5). This classification includes a central memory CX3CR1^NEG^ population that expresses higher levels of L-selectin and is more prevalent in the lymphoid tissue, a CX3CR1^INT^ population that expresses lower levels of L-selectin and circulates between the peripheral tissue and the blood, and a CX3CR1^HI^ population that lacks L-selectin and the migratory capacity of the other populations and is predominantly effector memory/terminally differentiated in phenotype (5). CX3CR1^INT^ CD8^+^ T cells have been described as peripheral memory T cells (5). They undergo more homeostatic divisions than any other memory subset to maintain self-renewal and can also convert to CX3CR1^NEG^ to feed the central memory pool of CD8^+^ T cells (5). However, the trigger or mediators for such transition have not been previously identified. To date, few studies have looked at the tissue localization of CX3CR1^+^ CD8^+^ T cell populations with recent reports of their migration to lymph nodes, spleen, bone marrow, lung, and liver in murine models of viral infection (5, 29). Here, treatment of peripheral blood CD8^+^ T cells with the CX3CR1 ligand fractalkine resulted in significant reduction in their CX3CR1 surface expression and thus expanded the CX3CR1^NEG^ pool of CD8^+^ T cells. Due to the abundance of fractalkine in the omentum and due to previous reports that CX3CR1^INT^ CD8^+^ T cells can replenish the CX3CR1^NEG^ CD8^+^ T cell pool, we propose that fractalkine recruits circulating T cells to omentum and subsequently mediates the conversion from CX3CR1^INT^ CD8^+^ T cells to CX3CR1^NEG^ CD8^+^ T cells, thus implicating the omentum as a key site in the accumulation of the memory CD8^+^ T cell pool in EAC and potentially other conditions. While CX3CR1 is the specific receptor for fractalkine, it must be noted that Eotaxin-3 (CCL26) has been identified as a functional chemoattractant for CX3CR1^+^ CD8^+^ T cells (30). However, our data have revealed that EAC omentum is not an abundant source of Eotaxin-3 (data not shown), compared to chemokines such as MIP-1α and fractalkine thus reducing the likelihood of this chemokine governing CX3CR1^+^ CD8^+^ T cell trafficking to omentum in EAC.

### Fractalkine Mediates CX3CR1^INT^ to CX3CR1^NEG^ Conversion in Peripheral Blood-Derived CD8^+^ T Cells *via* Receptor Endocytosis

CX3CR1 has recently been identified as a marker of CD8^+^ T cell memory during viral infection in murine models but the role if any of fractalkine in the differentiation of such T cells has not been described (5). Here, a significant decrease in CX3CR1 surface expression following fractalkine treatment was observed in the CD8^+^ T cell compartment in the peripheral blood but not in the T cell populations containing lower levels of CX3CR1 expression; circulating CD4^+^ T cells or omental CD4^+^ or CD8^+^ T cells. Such cell type- and compartment-specific cell surface regulation of a chemokine receptor is not unique to CX3CR1 and similar findings have been reported with CXCR4 (31). Indeed, the internalization of CXCR4 facilitated its association with the T cell receptor and enhanced co-stimulation of cytokine production by T cells thus emphasizing the multifaceted functions of chemokines in immunity (3). Further work performed in this study has revealed that the reduction in CX3CR1 expression is due to endocytosis in line with previous reports on macrophages in sepsis patients (32). To our knowledge, this is the first report of CX3CR1 endocytosis on CD8^+^ T cells following ligand binding and implicates fractalkine as master regulator of CX3CR1^INT^ to CX3CR1^NEG^ conversion. Such endocytosis is not followed by CX3CR1 recycling to surface within 48 h of ligand binding even when fractalkine is removed. Furthermore, fractalkine-mediated endocytosis of its receptor does not lead to increased levels of CX3CR1 shedding. In fact, our western blot data show that the protein accumulates within the cell and stimulation with an array of inflammatory cytokines simulating the soluble cues of an inflammatory microenvironment could not trigger its recycling to the cell surface. These data suggest that fractalkine elicits significant and long-lasting changes in CD8^+^ T cells, which may culminate in their retention in the omentum.

### Enhanced L-Selectin Expression by Peripheral Blood-Derived CD8^+^ T Cells Following Fractalkine-Mediated Conversion From a CX3CR1^INT^ to a CX3CR1^NEG^ Phenotype

An abundance of soluble ICAM-1 and L-selectin levels were detected in the omentum-derived ACM of EAC patients, suggesting their shedding by cells in this inflamed tissue and may be indicative of their role in immune cell arrest and retention in the VAT. Significant higher frequencies of ICAM-1^+^ and L-selectin^+^ cells within the CX3CR1^NEG^ CD8^+^ T cell population are also reported here. Since CX3CR1^NEG^ CD8^+^ T cells are the predominant subset within the omentum, our data suggest that such cells have altered adhesive and migratory properties and this may serve a function in retention within the omentum. Interestingly, our data also revealed significantly different memory phenotypes between the peripheral blood CX3CR1^NEG^ and CX3CR1^INT^ CD8^+^ T cell subsets and such characteristics have been previously described together with differences in their migratory properties and those of CX3CR1^HI^ CD8^+^ T cells (5). Others have shown that CX3CR1^INT^ CD8^+^ T cells in mice can increase their L-selectin expression and convert to CX3CR1^NEG^ CD8^+^ T cells, unlike CX3CR1^HI^ CD8^+^ T cells (5). For the first time, we identify fractalkine as a mediator of increased L-selectin expression by peripheral blood CD8^+^ T cells following their fractalkine-mediated conversion from a CX3CR1^INT^ to CX3CR1^NEG^ phenotype and we propose that these sequenced alterations occur in the fractalkine-rich environment of the omentum changing the migratory properties of CD8^+^ T cells in EAC. While soluble fractalkine induces changes in L-selectin surface expression, that of ICAM-1, VLA-4, LFA-1, alpha-4, and beta-integrin together with memory phenotype are unchanged. Contrary to ICAM-1 expression being highest within the CX3CR1^NEG^ CD8^+^ T cell population in blood, these data reveal that fractalkine-mediated conversion of CX3CR1^INT^ to CX3CR1^NEG^ CD8^+^ T cells is not accompanied by an increase in ICAM-1 expression and this event may be mediated by another soluble factor. These data identify a novel role for fractalkine in the alteration of migratory and lymphoid tissue homing capacity of CD8^+^ T cells and align with previous reports of fractalkine and CX3CR1 regulating immune cell responses *via* distinct disease-dependent mechanisms (28, 32). For instance, this chemokine axis promotes T cell survival in asthma, while it promotes T cell retention in inflamed skin in dermatitis and plays a role in immune paralysis in sepsis (11, 28, 32). However, for the first time, this study identifies fractalkine as a player in T cell recruitment to omentum and a master regulator of CX3CR1 expression, CX3CR1^INT^ to CX3CR1^NEG^ conversion and L-selectin expression. Together with our previously published data, we propose that such alterations change the fate of CD8^+^ T cell trafficking and CD8^+^ T cell-mediated immunity in EAC and may lead to their retention in the fractalkine-rich omentum where they contribute to pathological inflammation to the detriment of effective anti-tumor immunity.

## Conclusion

Fractalkine-driven migration of T cells to omentum in EAC is likely to contribute to the CD8^+^ T cell-mediated adipose tissue inflammation previously demonstrated in these patients (22, 23). In pilot data not shown, we have also found lower levels of soluble fractalkine in tumor compared to omentum in our patient cohort. Since the fractalkine:CX3CR1 pathway has been shown to have a selective bias for cytotoxic lymphocytes such as CD8^+^ T cells and NK cells, the preferential migration of CD8^+^ T cells to omentum over tumor might be detrimental for anti-tumor immunity in EAC and other obesity-associated cancers (33). Antagonizing the CX3CR1:fractalkine pathway might attenuate CD8^+^ T cell-mediated inflammation in the omentum but secondary to this it might prevent the CX3CR1^INT^ to CX3CR1^NEG^ conversion and disrupt frequencies and migration of peripheral and central memory CD8^+^ T cells, which would also be detrimental to anti-tumor immunity. Therefore, our findings place fractalkine as more than a chemotactic cytokine driving T cell-mediated inflammation in EAC. Further *in vivo* work will be necessary to elucidate the consequences of blocking this pathway in obesity-associated cancer. These novel data advance our knowledge on the multifaceted functionality of fractalkine and may inform T cell therapies and chemokine-targeted therapies in the future.

## Author Contributions

MC obtained Irish Research Council funding; performed the acquisition, analysis, and interpretation of data; drafted and finalized the manuscript; completed the statistical analysis; and drove study concept and design. SM performed the acquisition, analysis, and interpretation of data. SD acquired data. AM, EF, and JR provided material support. AL provided technical support and performed a critical revision of the manuscript for intellectual content. JL obtained Health Research Board funding and provided technical and material support, drove study concept and design, and performed a critical revision of the manuscript for intellectual content.

## Conflict of Interest Statement

The authors declare that the research was conducted in the absence of any commercial or financial relationships that could be construed as a potential conflict of interest.
